# Another Victim to Quackery

**Published:** 1848-12

**Authors:** 


*Another Victim to Quackery.*—In one of the Western Medical Journals, Dr. Meeker, of Laporte, Indiana, details a case of cancer or scirrhus tumor in the breast, to which a "*cancer doctor*" first applied a blister and removed the cuticle, and then a plaster spread over with a fine white powder. The plaster occasioned great pain, but was continued on the breast eleven hours. After it had been on only two hours, the patient began to feel a metallic taste in the mouth, nausea, griping pains in the stomach and bowels; and soon after vomiting with spasmodic action of the muscles. These symptoms continued until the plaster was removed, when a dose of laudanum was given, and a physician sent for. He came in a few hours, and found the patient as follows, viz: "Pulse 126, violent spasms, constriction of the muscles of the pharynx, burning sensation in the œsophagus, nausea and vomiting, cold extremities." &c. She lingered until the seventh day, and died, notwithstanding the most judicious and faithful attention. Dr. Meeker was called in consultation, and finding the "cancer doctor" in the house, induced him to state the composition of the powder; which was *corrosive sublimate,* in quantity nearly 100 grains. He alledged that "he had applied it to a great number of cancers, but never knew it to produce such an effect before."

After the fatal result, the old quack explained it all, by saying "that the roots of the cancer *reached the heart,* and that the *medicine,* in following them out, produced death." Some, however, not seeming satisfied with this explanation, he secured his own safety by decamping with twenty dollars, which he received, in part pay, for the murder. The rapidity with which the poison was absorbed in this case is worthy of notice.—*Annalist.*

